# Confident Learning-Based Label Correction for Retinal Image Segmentation

**DOI:** 10.3390/diagnostics15141735

**Published:** 2025-07-08

**Authors:** Tanatorn Pethmunee, Supaporn Kansomkeat, Patama Bhurayanontachai, Sathit Intajag

**Affiliations:** 1 Division of Computational Science, Faculty of Science, Prince of Songkla University, Songkhla 90110, Thailand; 2 Department of Ophthalmology, Faculty of Medicine, Prince of Songkla University, Songkhla 90110, Thailand

**Keywords:** labeling error, label noise correction, confident learning, semantic segmentation, retinal images, dataset refinement, pixel-level annotation, medical image segmentation

## Abstract

**Background/Objectives:** In automatic medical image analysis, particularly for diabetic retinopathy, the accuracy of labeled data is crucial, as label noise can significantly complicate the analysis and lead to diagnostic errors. To tackle the issue of label noise in retinal image segmentation, an innovative label correction framework is introduced that combines Confident Learning (CL) with a human-in-the-loop re-annotation process to meticulously detect and rectify pixel-level labeling inaccuracies. **Methods:** Two CL-oriented strategies are assessed: Confident Joint Analysis (CJA) employing DeeplabV3+ with a ResNet-50 architecture, and Prune by Noise Rate (PBNR) utilizing ResNet-18. These methodologies are implemented on four publicly available retinal image datasets: HRF, STARE, DRIVE, and CHASE_DB1. After the models have been trained on the original labeled datasets, label noise is quantified, and amendments are executed on suspected misclassified pixels prior to the assessment of model performance. **Results:** The reduction in label noise yielded consistent advancements in accuracy, Intersection over Union (IoU), and weighted IoU across all the datasets. The segmentation of tiny structures, such as the fovea, demonstrated a significant enhancement following refinement. The Mean Boundary F1 Score (MeanBFScore) remained invariant, signifying the maintenance of boundary integrity. CJA and PBNR demonstrated strengths under different conditions, producing variations in performance that were dependent on the noise level and dataset characteristics. CL-based label correction techniques, when amalgamated with human refinement, could significantly enhance the segmentation accuracy and evaluation robustness for Accuracy, IoU, and MeanBFScore, achieving values of 0.9156, 0.8037, and 0.9856, respectively, with regard to the original ground truth, reflecting increases of 4.05%, 9.95%, and 1.28% respectively. **Conclusions:** This methodology represents a feasible and scalable solution to the challenge of label noise in medical image analysis, holding particular significance for real-world clinical applications.

## 1. Introduction

Automatic medical image analysis has emerged as a cornerstone of modern healthcare diagnostics, with retinal imaging serving as a critical tool for the early detection and monitoring of various ocular and systemic diseases [1]. The segmentation of retinal structures, particularly blood vessels, plays a vital role in diagnosing conditions such as diabetic retinopathy (DR), hypertension-related retinopathy, and glaucoma [2,3]. As these conditions represent leading causes of preventable blindness globally, the development of accurate and reliable automated segmentation systems has become increasingly important for supporting clinical decision-making and enabling large-scale screening programs [4,5].

Deep learning approaches have revolutionized retinal image segmentation, with convolutional neural networks (CNNs) demonstrating remarkable performance in extracting complex vascular patterns [6,7,8]. However, the success of these models is fundamentally dependent on the quality of the training data labeling. The accurate annotation of medical images is particularly challenging due to the intricate nature of anatomical structures. The task is further complicated by interobserver variability and the substantial time and expertise required for manual annotation. Consequently, even widely used benchmark datasets may contain labeling errors that can significantly impact model training and evaluation [9,10,11,12,13,14,15].

The segmentation challenge presented by label noise in retinal image datasets extends beyond simple annotation errors. Pixels may be mislabeled due to ambiguous vessel boundaries, poor image quality, artifacts, and the subjective interpretation of complex vascular structures. These labeling inaccuracies can lead to several detrimental effects: (1) the introduction of poor outcomes during model training, as networks learn incorrect patterns; (2) unreliable performance evaluation due to errors in test datasets; and (3) potential misdiagnoses when precision is paramount [16,17,18,19,20,21].

Recent studies have highlighted the pervasive nature of labeling errors in medical imaging benchmarks, revealing that even well-established datasets used for algorithm development and validation may contain significant annotation inconsistencies [20,22]. This realization has prompted a re-evaluation of traditional approaches to model training and performance assessment, emphasizing the need for robust methods that identify and correct labeling errors systematically.

To address this challenge, we propose a novel framework that leverages Confident Learning (CL), a principled approach for characterizing and correcting label noise in datasets [23,24,25]. CL provides a theoretical foundation for estimating the joint distribution of noisy and true labels, enabling the identification of potentially mislabeled examples with statistical confidence. By integrating CL methodologies with human re-annotation processes, our approach aims to enhance both the quality of training data and the reliability of model evaluation in retinal image segmentation tasks.

This study makes several key contributions to the field of medical image analysis. First, we have developed an effective and scalable label correction framework that combines CL techniques with non-expert human verification, making it accessible in practical clinical applications. Second, we have implemented and evaluated two distinct CL-based correction strategies—Confident Joint Analysis (CJA) and Prune by Noise Rate (PBNR)—using different neural network architectures to assess their effectiveness across various noise conditions. Third, we have provided comprehensive experimental evidence that correcting labeling errors, particularly in validation datasets, significantly improves the accuracy and reliability of performance assessments. Finally, we have conducted a cross-validation analysis between the two methodologies to evaluate their consistency and potential for synergistic application.

The remainder of this paper is organized as follows: Section 2 reviews related works in retinal image segmentation, label noise handling, and CL approaches. Section 3 details our proposed methodology, including the CL-based correction strategies and implementation details. Section 4 presents the experimental results obtained from four public retinal image datasets. Discussions of the results and study limitations follow in Section 5 and Section 6. Section 7 presents our conclusions.

## 2. Related Works

### 2.1. Retinal Image Segmentation

Retinal image segmentation is a fundamental task in ophthalmic medical image analysis. Early approaches relied on traditional image processing techniques such as matched filtering, morphological operations, and thresholding methods [4,5]. The advent of machine learning introduced supervised methods utilizing hand-crafted features, with ensemble classifiers showing promising results [11]. However, these approaches often struggled with the complex and variable nature of the retinal vasculature, particularly in pathological cases. More recently, Hao et al. [26] proposed an uncertainty-aware iterative learning framework that effectively handled noisy labeled retinal images, demonstrating improved segmentation performance in challenging scenarios.

The introduction of deep learning, particularly fully convolutional networks, marked a paradigm shift in retinal image segmentation [6]. Long et al. pioneered their use for semantic segmentation, demonstrating that end-to-end learning could capture complex spatial relationships without manual feature engineering. Tan et al. [27] aimed for a broader segmentation of the optic disc, the fovea, and vessels using a single, unified network, potentially trading some specialization for comprehensiveness and efficiency. Various architectural innovations have since been proposed for retinal vessel segmentation. Alsayat et al. [28] introduced a framework for segmenting blood vessels in retinal images that employed a multi-layer preprocessing stage and a segmentation stage using a U-Net with a multi-residual attention block. Liu et al. [29] proposed a framework for automatic retinal vessel segmentation using a three-path U-Net model, which enhanced segmentation performance by integrating multi-scale contextual information. Their method demonstrated strong performances on benchmark datasets such as DRIVE and CHASE_DB1. Li et al. [7] introduced Dense-U-Net, which leveraged dense connections to improve gradient flow and feature reuse. DeepLabV3+, proposed by Chen et al. [8], incorporated atrous spatial pyramid pooling and encoder–decoder structures to achieve state-of-the-art performances on multiple benchmarks.

Despite these advances, the performance of deep learning models remains fundamentally limited by the quality of the training data. The established benchmark datasets for retinal vessel image segmentation include DRIVE [9], STARE [10], CHASE_DB1 [11], and HRF [12]. More recent datasets such as FIVES [13], RETA [14], and MAPLES-DR [15] have expanded the available resources. However, these datasets rely on manual annotations that introduce interobserver variability and may contain errors due to the inherent difficulty of delineating fine vascular structures.

### 2.2. Label Noise in Medical Image Segmentation

The impact of label noise on machine learning models has been recognized since the foundational work of Angluin and Laird [23], who demonstrated that learning algorithms could tolerate random classification noise under certain conditions. In the context of medical image segmentation, label noise presents unique challenges due to the continuous nature of segmentation masks and the critical importance of accuracy in clinical applications.

Guo et al. [16] specifically discussed medical image segmentation with noisy labels, focusing on imbalanced data and pixel-dependent noise. Their focus on the degradation of segmentation models by label noise led to the development of a machine learning framework that was stronger and more domain-specific than frameworks that were focused more on annotation improvements, semi-supervised methods, or uncertainty calibration. Pranto et al. [17] extended this analysis to multi-class segmentation tasks, demonstrating that certain classes were more susceptible to label noise than others. In medical imaging specifically, Xu et al. [18] proposed a mean-teacher-assisted approach for learning from noisy labels, leveraging consistency regularization to improve robustness.

Tajbakhsh et al. [19] provided a comprehensive review of deep learning solutions for segmenting medical images from imperfect datasets, categorizing the approaches into noise-robust architectures, loss functions, and data cleaning methods. They highlighted that most existing methods focused more on handling noise during training rather than correcting the labels themselves. Northcutt et al. [20] made a significant contribution by revealing pervasive label errors in popular test sets, demonstrating that benchmark instability could lead to incorrect conclusions about model performances.

Karimi et al. [21] specifically addressed deep learning with noisy labels, proposing noise modeling, robust loss functions, and curriculum learning techniques for medical image analysis. However, they noted that most approaches assumed access to a small clean dataset or knowledge of the noise distribution, which may not be realistic in practice. More recently, Hao et al. [22] proposed an uncertainty-aware iterative learning framework for noisy labeled medical image segmentation, highlighting the importance of robust methods to detect and mitigate label noise.

### 2.3. Confident Learning and Label Correction

CL was introduced by Northcutt et al. [24,25] to provide a principled framework for identifying and correcting labeling errors in datasets. Unlike traditional noise-handling approaches that adapt model training to accommodate errors, CL directly estimates the joint distribution between noisy and true labels, enabling the identification of likely mislabeled examples.

The theoretical foundation of CL rests on three key principles: (1) pruning, which identifies and removes noisy examples; (2) counting, which estimates confident predictions to characterize class-conditional label noise; and (3) ranking, which orders examples by the likelihood of being mislabeled. This framework has been successfully applied to classification tasks but requires adaptation for segmentation problems where the labels are pixel-wise rather than image-wise.

Lad and Mueller [30] extended CL concepts to semantic segmentation, proposing methods to estimate label quality and errors at the pixel level. Their approach demonstrated that CL principles could be adapted to dense prediction tasks, though computational challenges arise due to the increased complexity of pixel-wise predictions. Zhang et al. [31] addressed the challenge of disentangling human error from ground truth in medical image segmentation, proposing a probabilistic model that separately estimates annotator reliability and true segmentation masks.

In the context of segmenting medical images with noisy labeling, Zhang et al. [32] characterized label errors using CL principles, developing methods to identify and correct mislabeled pixels. Shi and Wu [33] proposed distilling effective supervision from noisy labels, combining CL concepts with knowledge distillation to improve segmentation robustness. Recently, Weng et al. [34] demonstrated the integration of superpixel methods and CL to guide the improvement of point-based annotations in semi-supervised OCT fluid segmentation. Their approach highlighted the adaptability of CL principles to medical segmentation tasks that require precise localization and label correction, which are highly relevant to the challenges in retinal image segmentation.

## 3. Materials and Methods

Our proposed framework integrates CL principles with human re-annotation to systematically identify and correct label errors in retinal image segmentation datasets. The methodology consists of five main components: (1) materials, (2) network architectures, (3) a CL-based pixel-level label noise estimation, (4) the implementation of two distinct CL strategies—Confident Joint Analysis (CJA) and Prune by Noise Rate (PBNR)—to compare their effectiveness across different architectures and noise conditions, and (5) evaluation metrics.

### 3.1. Materials

In order to assess the efficacy of our proposed methodology, we executed a series of experiments employing four publicly accessible retinal image datasets: DRIVE, STARE, CHASE_DB1, and HRF. Collectively, these datasets encompass a total of 133 images, each paired with an expert-annotated segmentation mask. The specifics of each dataset can be found in Table 1. The datasets were chosen for their variability in image quality and annotation style. The DRIVE and CHASE_DB1 datasets served as the foundational basis for initializing the network models utilized to investigate and explore noisy identification within the STARE and HRF datasets. All images underwent a resizing process to achieve a resolution of 1024 × 1024 pixels through the application of the nearest neighbor interpolation method.

**Figure 1:**
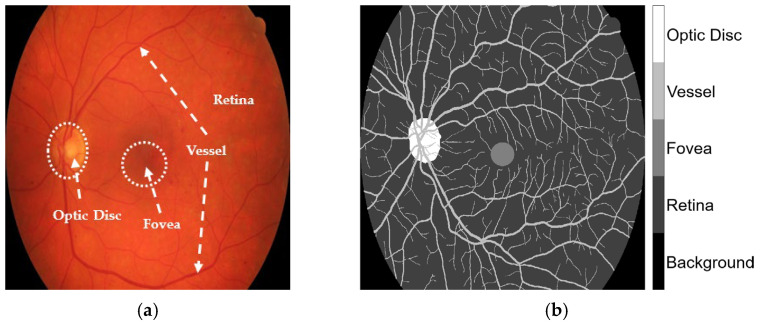
Example from the HRF dataset: (**a**) original retinal image, Image1024-02_g; and (**b**) corresponding ground truth annotation.

The HRF, DRIVE, and CHASE_DB1 datasets facilitated the examination of label inaccuracies within the CLA framework. The ground truths were first modified by incorporating three additional classes, retina, optic disc, and fovea, as shown in Figure 1. The revised annotations were conducted under the guidance of seasoned ophthalmologists who delivered precise delineations to guarantee accuracy in the segmentation methodology. When these three classes were overlaid, they became major anatomic structures in the retinal images. Blood vessels in the STARE database were manually annotated by two experts, Adam Hoover (AH) and Valentina Kouznetsova (VK), as referenced in [10]. The annotated data are publicly available at https://cecas.clemson.edu/~ahoover/stare/probing/index.html (accessed on 2 July 2025). These annotations are used as reference labels to evaluate label rectification within the PBNR framework.

### 3.2. Network Architecture

DeepLabV3+ is a state-of-the-art semantic segmentation model that integrates various components from previous models to improve performance. It employs an encoder-decoder structure, where the encoder extracts high-level features from input images using a backbone network, while the decoder refines these features for precise segmentation. A key feature of DeepLabV3+ is the use of atrous, or dilated, convolutions and atrous spatial pyramid pooling (ASPP). The atrous convolutions expand the receptive field without increasing the number of parameters. This allows the model to capture multiscale contextual information effectively, which is essential for segmenting the optic disc, fovea, and vessels of varying sizes. The ASPP module performs parallel atrous convolutions at different rates, enabling the model to gather the global context and improve the segmentation accuracy. This is particularly beneficial for retinal images, where vessels can be thin and intricate.

To examine the segmentation of anatomic structures of the retina and rectify labeling discrepancies in blood vessel datasets, two foundational architectures—ResNet [35] and DeepLabV3+—were deployed. This approach facilitated a thorough assessment of segmentation efficacy in different conditions and datasets. The foundational model, ResNet50, is a sophisticated deep convolutional neural network that employs residual connections, thereby enhancing the training of deeper architectures. It allows DeepLabV3+ to capture intricate multiscale contextual information through atrous convolutions, culminating in accurate anatomic structure segmentation. Its proficiency in representing complex features is particularly beneficial in challenging scenarios characterized by label noise and intricate structures, and enabled the investigation of the CJA framework, designated as CJA-RN50DLv3+. Conversely, the ResNet-18 backbone is a more superficial and lightweight residual network, optimized for expedited inference and diminished computational requirements. It was employed to encode within DeepLabV3+ specifically for the segmentation of blood vessels, which facilitated the exploration of PBNR frameworks, referred to as PBNR-RN18DLv3+.

### 3.3. CL-Based Pixel-Level Label Noise Estimation

CL is a technique for identifying and correcting labeling errors by analyzing the agreement between model predictions and observed labels using the predicted class probabilities of a model as evidence. It systematically detects instances where the predicted label likely differs from the true label, enabling an accurate refinement of the annotation. The CL-based method is particularly effective in medical imaging and was developed for label refinement in two strategies: CJA and PBNR. CJA estimates the joint distribution of true and noisy labels using a confident joint matrix. This operation allows the identification of mismatches where the model is confident that the observed label is incorrect. PBNR ranks samples based on their likelihood of being mislabeled and selectively prunes or flags low-confidence samples using noise rate estimates to reduce the influence of label noise. Both methods, CJA and PBNR, follow a shared sequence of key steps.

1. Computing Confidence Scores

A semantic segmentation model was trained using noisy labels (ỹ) obtained from non-expert annotations. After training, the CJA-RN50DLv3+ and PBNR-RN18DLv3+ frameworks generated class-wise predicted probabilities for each pixel *x* ∈ *X*, denoted as $\hat{p}(\tilde{y}=i;\;x,\;\theta )$, where *i* is the class index and *θ* represents the model parameters.

2. Identifying Noisy Labels

For the CJA-RN50DLv3+ model, the confidence scores were used to estimate the likelihood that each pixel was correctly labeled. By comparing these confidence scores with the original labels, potential label errors could be inferred. For the PBNR-RN18DLv3+ model, label noise was modeled using a noise transition matrix to indicate the probability of one class being mislabeled as another. Two annotation sets from the STARE dataset—AH and VK—are used in this study. The AH set was treated as a set of naturally observed labels, while the VK set of annotations served as the ground truth. The noise transition matrix was defined as

(1) $$T_{\tilde{y},\;y*}=P(\tilde{y}\;=\;i\;|\;y*=j)$$

where

- T_ỹ, y*_ denotes the noise transition matrix;
- *ỹ* is the observed noisy label;
- y* is the unobserved, latent, or correct label.
- *P*(*ỹ* = i | y* = *j*) is the conditional probability that a true label y* = *j* is flipped to a noisy label *ỹ* = *i*.

3. Computing Class-Wise Confidence Thresholds

For every categorized class, a confidence threshold, $t_{j}$, was determined to differentiate true positives from false positives as follows:

(2) $$t_{j}=\frac{1}{\left|X_{\tilde{y}\;=j}\right|}\sum_{x\in X_{\tilde{y}\;=j}}\hat{p}(\tilde{y}\;=j;\;x,\;\theta )$$

In Equation (2),

- $t_{j}$ represents the confidence threshold for class j;
- $X_{\tilde{y}\;=j}$ is the set of pixels originally labeled as class j.
- $\hat{p}(\tilde{y}\;=j;\;x,\;\theta )$ is the model-predicted probability of pixel x belonging to class j.

4. Constructing the Confident Joint Matrix

The confident joint matrix, denoted as $C_{\tilde{y},\;y*}$, quantified the relationship between noisy labels (*ỹ*) and inferred true labels (y*). Each entry $C_{\tilde{y},\;y*}[i][j]$ represented the number of pixels labeled as class *i* in the noisy dataset, and confidently predicted by the model to belong to class *j*, based on class-specific confidence thresholds.

Confidence was determined by comparing the model-predicted probability $\hat{p}(\tilde{y}\;=j;\;x,\;\theta )$ to a threshold $t_{j}$ for class *j*, typically estimated as the mean predicted probability of that class. The confident joint matrix was formally defined as

(3) $$\begin{array}{c} C_{\tilde{y},\;y*}[i][j]\;≔\;|{\hat{X}}_{\tilde{y}=i,\;y*=j}|,\;\mathrm{where}\\ {\hat{X}}_{\tilde{y}=i,\;y*=\;j}\;≔\;\{x\;\in {\;X}_{\tilde{y}=i}\;:\;\hat{p}(\tilde{y}\;=j;\;x,\;\theta )\;\geq \;t_{j},\;j\;=\underset{l\;\in \;\left[m\right]:\hat{p}(\tilde{y}\;=l;\;x,\theta )\;\geq \;t_{l}}{\mathrm{argmax}}\hat{p}(\tilde{y}\;=l;\;x,\;\theta )\} \end{array}$$

in which

- ${\hat{X}}_{\tilde{y}=i{,y}^{*}=j}$ is the set of samples labeled as class *i* but are inferred to belong to class *j*, based on the confidence threshold $t_{j}$;
- $\hat{p}(\tilde{y}\;=j;\;x,\;\theta )$ denotes the model-predicted probability that sample *x* belongs to class *j*, given parameter *θ*;
- $t_{j}$ is the confidence threshold for class *j*;
- The argmax selects the class l ∈ [m] with the highest predicted probability among those whose predicted probabilities exceed their respective thresholds $t_{l}$. This operation ensures that the inferred class label *j* is both confident and the most probable among the eligible classes.

5. Computing the Normalized Margin-Based Pixel Ranking

To prioritize pixels most likely to be mislabeled in the PBNR approach, we computed the normalized margin for each observed label. This margin quantified the confidence gap between the predicted probability of the noisy label *ỹ* and the highest predicted probability across all other classes. The normalized margin was defined as

(4) $$\mathrm{Normalized}\;\mathrm{Margin}=\frac{\left(p-p_{\max}\right)+1}{2}$$

where

- *p* is the predicted probability assigned to the noisy label *ỹ*;
- $p_{\max}$ is the maximum predicted probability among the remaining classes.

Pixels were then ranked in ascending order based on their normalized margin scores. A lower normalized margin indicated greater uncertainty, and hence, a higher likelihood of mislabeling.

In the traditional PBNR method, the lowest-ranked pixels—corresponding to the estimated noise rate per class—would typically be removed from the training dataset. However, in this study, we retained all samples. Rather than potentially discarding useful data, we used the normalized margin ranking to guide label refinement, thereby maintaining dataset integrity while still mitigating the impact of label noise.

### 3.4. Implemented Frameworks

**Figure 2:**
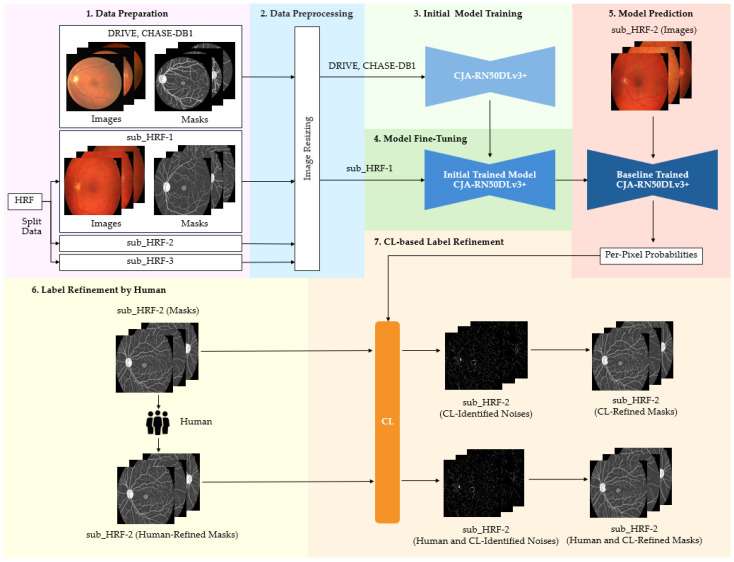
Procedural framework of CL-based label refinement employing the CJA method. CJA discerns inaccurately labeled pixels through model predictions and facilitates automated label rectification, ultimately augmenting the quality of annotations and the efficacy of image segmentation.

**Figure 3:**
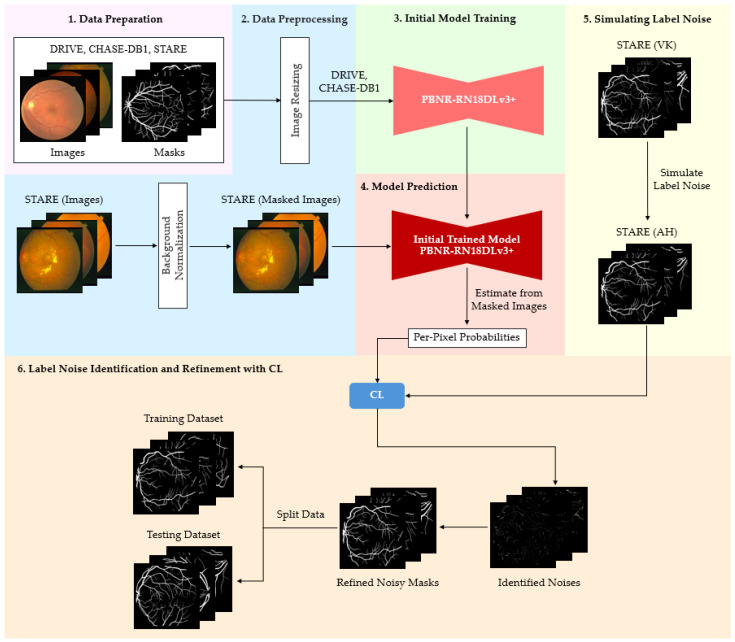
Procedural framework of label noise identification and refinement utilizing CL through the PBNR method. PBNR detects inaccurately labeled pixels by assessing class-dependent noise rates derived from model predictions, subsequently eliminating noise based on these assessments. When integrated with CL, this approach permits the automated refinement of labeling used in training, thereby enhancing the annotation quality and bolstering the resilience of image segmentation.

The models utilized for the implementation of label correction via CJA and PBNR are illustrated in Figure 2 and Figure 3, respectively. The methods put forward not only augment the precision of segmentation but also establish a resilient framework for mitigating label noise within medical imaging datasets. The methods are described below.

#### 3.4.1. CJA Model

CJA constitutes a fundamental methodology within the CL framework that is specifically designed to identify and rectify labeling inaccuracies within machine learning datasets. This technique discerns likely instances of mislabeling by computing the confident joint matrix, as in Equation (3), which encapsulates the joint distribution between authentic labels and the observed labels from the datasets. To evaluate the contribution of CJA-driven label refinement to enhanced image segmentation performance, CJA-RN50DLv3+ was deployed to study, evaluate, and correct the labeling in the HRF dataset. The CJA framework is depicted in Figure 2. The incorporation of CL into the training framework was anticipated to augment the quality of the training dataset and consequently enhance the segmentation results. The subsequent subsections provide a detailed account of each phase of the CJA workflow.

**Figure 4:**
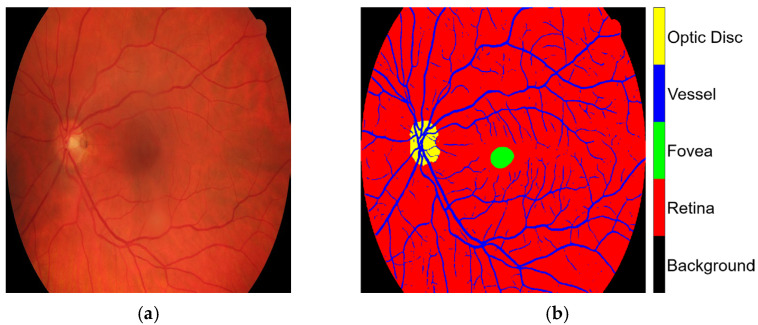
Prediction results from the calibrated CAJ-RN50DLv3+ model: (**a**) original retinal image, Image1024-02_g; and (**b**) predicted segmentation mask showing five categories—optic disc, vessels, fovea, retina, and background.

The following procedure is outlined in Figure 2. The modified datasets, DRIVE and CHASE-DB1, underwent preprocessing to adjust the spatial resolution to 1024 × 1024 pixels through the application of nearest neighborhood interpolation on both the images and their corresponding masks. These datasets provided the training data for the CJA-RN50DLv3+ model, which was instrumental in initializing the segmentation of major anatomic structures of the retina. The obtained HRF training dataset was randomly partitioned into three subsets, with sub_HRF-1 and sub_HRF-2 each comprising ten images, while sub_HRF-3 comprised the remaining images. The subset sub_HRF-1 was utilized for the fine-tuning of the CJA-RN50DLv3+ model. The fine-tuned model was evaluated on sub_HRF-2 to determine its efficacy in accurately segmenting retinal structures. Some evaluative results are illustrated in Figure 4. These submodules facilitated the preparation of the model for the CJA process.

**Figure 5:**
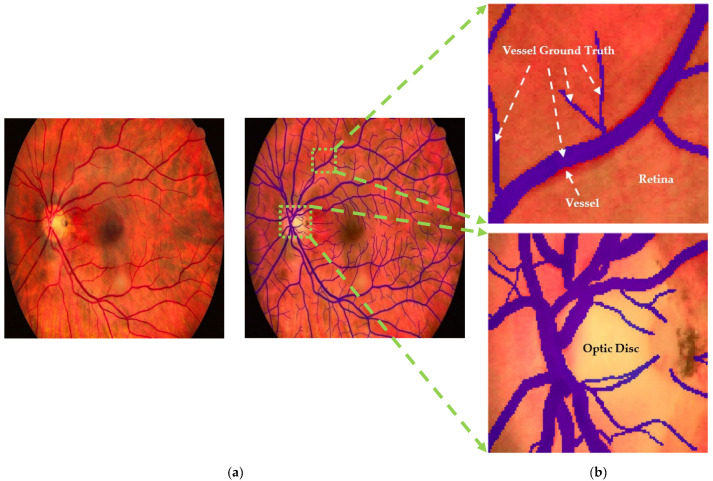
Illustration of the refinement procedure aimed at rectifying blood vessel delineations. (**a**) The original retinal image is enhanced through CLAHE and subsequently overlaid with the ground truth to rectify instances of false positives and false negatives. (**b**) A focused crop and magnification of the blood vessel and optic disc are employed to elucidate enhancements omitted from the ground truth of the vascular region (false negative); furthermore, certain areas of the retinal region that the ground truth mistakenly identifies as blood vessels (false positive) are rectified to correspond to the retinal class.

The CJA process, as described in Section 3.3, was then implemented. The confidence threshold for each $t_{j}$ category was computed from Equation (2) (Table 2). In order to construct the confident joint matrix for the entire set of images within the sub_HRF-2 dataset, it was imperative to examine the original ground truths. The original images underwent enhancement utilizing Contrast Limited Adaptive Histogram Equalization (CLAHE) [36] to augment the anatomic details of the retina, and the Image Labeler application within MATLAB [37] was used to rectify labeling inaccuracies present in each class, as depicted in Figure 5. These meticulously refined ground truths facilitated the calculation of the confident joint matrix from Equation (3), as demonstrated in Table 3.

**Figure 6:**
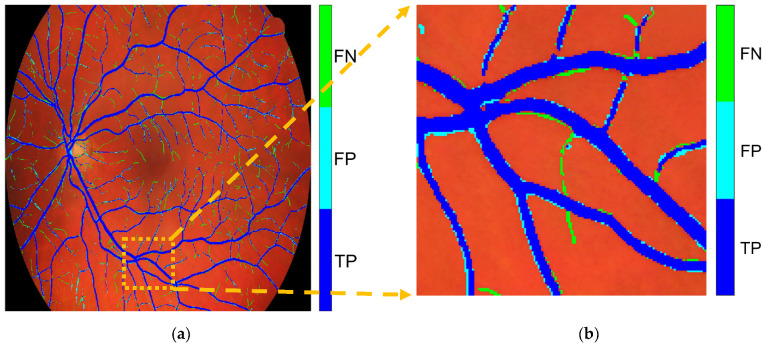
Segmented results of Image1024-02_g: (**a**) overlay with true positives (TP), false positives (FP), and false negatives (FN) for the vessel class; and (**b**) zoomed-in regions for a detailed inspection using the original masks.

Figure 6 visualizes the segmentation results specifically for the vessel class, illustrating the relationships in the corresponding row and column of the confident joint matrix shown in Table 3. The dark blue region represents true positive (TP) pixels—those originally labeled as vessel (*ỹ*) and also confirmed as vessel by CL (y* = vessel). This TP region contained 81,780 pixels, where the predicted probability score for the vessel class exceeded the threshold of 0.9744 indicated in Table 2. The light blue region represents false positive pixels—originally labeled as other classes but predicted by CL as a vessel. These pixels included 4 labeled as background, 14,137 as retina, and 669 as optic disc. These 14,810 pixels exceeded the vessel threshold of 0.9744 but fell below the thresholds of their putative classes, indicating potential label noise. Conversely, the light green region represents false negative pixels—originally labeled as vessel but identified by CL as other classes. This group included 1 pixel now reclassified as background, 16,686 as retina, 1 as fovea, and 615 as optic disc. For these 17,303 pixels, the thresholds of their reclassifications exceeded the probability threshold for the vessel classification, suggesting mislabeling in the original annotation.

As illustrated in Figure 2, following the identification of noise and the refinement of masks by CL, sub_HRF-2 contained two distinct sets: an initial set originating from the CL refinement, and a second set derived from human refinement. Both were utilized to train the CJA-RN50DLv3+ model in order to evaluate the efficacy of the CJA framework during the experimental procedures.

#### 3.4.2. PBNR Model

The PBNR framework is illustrated in Figure 3. The framework constitutes a systematic approach to the identification of noise. Inaccurately labeled pixels are identified by evaluating class-specific noise rates derived from model predictions. The most erroneous instances are eliminated based on these assessments. The CL methodology facilitates the automated enhancement of training labels, thereby improving the quality of annotations and fostering more resilient segmentation outcomes.

The following procedure is outlined in Figure 3. The modified datasets DRIVE and CHASE-DB1 underwent a rigorous preprocessing phase to recalibrate the spatial resolution to 1024 × 1024 pixels via the implementation of nearest neighbor interpolation on both the images and their associated masks. These datasets constituted the foundational framework for the training of the PBNR-RN18DLv3+ model dedicated to blood vessel segmentation. The model, once trained, was used to forecast vessel classification from the STARE dataset, which preserved the predicted probability scores, $\hat{p}(\tilde{y}\;=i;\;x,\;\theta )$, necessary for the computation of the threshold values from Equation (2) (Table 4). In Equation (1), the true label, y*, was substituted with the VK set, while *ỹ* was derived from the AH set within the STARE dataset. In this way, the noise transition matrix could undergo retraining. In this study, label noise was not artificially introduced but was derived from inherent inaccuracies in the manual annotations of the DRIVE dataset. Although the annotations were created by expert graders, they still contained inconsistencies, particularly where regions of low contrast, ambiguous vessel boundaries, or fine vascular structures occurred. Such imperfections are common in medical image datasets and reflect the subjective nature of manual labeling. Previous studies (e.g., Zhang et al. [31]; Xu et al. [18]) have highlighted that even datasets annotated by experts can exhibit variability and noise. This naturally occurring label noise provides a realistic setting for evaluating the robustness of Confident Learning (CL)-based label refinement methods. To elucidate the identification of noise within the PBNR model, the image file, im0001, with its corresponding ground truth from the AH set, its threshold value, and its predicted probability, were utilized to establish the CL confident joint matrix in Table 5.

**Figure 7:**
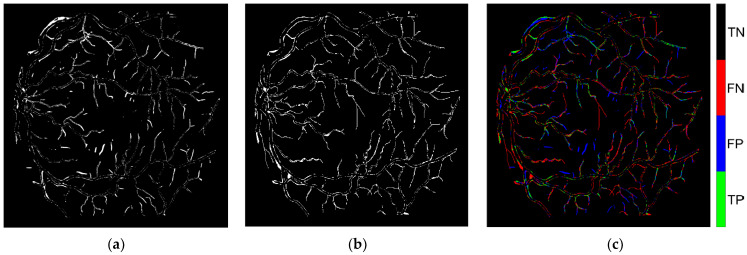
CL derivative pertaining to the image im0001: (**a**) the observed issue mask produced through the application of CL; (**b**) the authentic discrepancies identified between AH and VK; and (**c**) pixel-level visualization of TPs, FPs, FNs, and TNs between the observed and true issue masks.

The confident joint matrix shows for image im0001 shows 5981 false-positive pixels misclassified as background and 23,960 as a vessel. These values reflect the labeling discrepancies identified by the CL method and are visualized in the observed issue mask shown in Figure 7a.

To prioritize these potentially mislabeled pixels, the normalized margin, as defined in Equation (4), was applied to all pixels in the observed issue mask. Each pixel was then ranked in ascending order by its normalized margin score, where a lower score indicated higher uncertainty and a greater likelihood of being mislabeled.

To evaluate the effectiveness of CL in detecting label errors, PBNR was used to examine the overlap between Figure 7a (the observed issue mask) and Figure 7b (the true issue mask). Pixels present in both masks were classified as true positives (TPs) and highlighted in Figure 7c. Additional categories—false positives (FPs), false negatives (FNs), and true negatives (TNs)—were also represented in Figure 7c and summarized in Table 6. An FP refers to a pixel present in Figure 7a but not in Figure 7b, indicating an incorrectly flagged error. An FN refers to a pixel present in Figure 7b but absent from Figure 7a, representing an error missed by CL. A TN refers to a pixel not flagged by CL in Figure 7a and correctly labeled according to the ground truth, which is an appropriate case of no error detected.

Table 7 lists the top 10 candidate label errors in im0001, ranked by their normalized margin scores. Here, the “true label” refers to the VK ground truth annotation, while the “true issue” column indicates whether each candidate was a confirmed labeling error (one) or not (zero), based on a comparison with the true issue mask derived from annotation disagreements between AH and VK (Figure 7b).

As shown in Figure 3, following the detection of noise and the enhancement of masks through CL, the STARE ground truth identified certain observed pixels within the AH set, yielding refined observed masks and two subsets, one comprising fourteen images allocated for training, and the other comprising the remaining six images designated for testing. The training subset was used to instruct the PBNR-RN18DLv3+ model, with the objective of assessing the effectiveness of the PBNR framework throughout the experimental phase.

### 3.5. Evaluation Metrics

To evaluate the performance of the semantic segmentation models, we employed four widely-used metrics: accuracy (Acc), Intersection over Union (IoU), weighted IoU, and Mean Boundary F1 Score (MeanBFScore). These metrics capture complementary aspects of segmentation quality, particularly in the context of imbalanced medical datasets such as retinal vessel segmentation.

- Global Accuracy (Acc)

This metric evaluates the overall proportion of correctly predicted pixels across all classes. While it provides a general sense of model correctness, it may be less informative in scenarios with significant class imbalance, such as the dominance of background pixels over smaller structures like vessels. Acc was determined as follows:

(5) $$\mathrm{Acc}=\frac{\mathrm{TP}+\mathrm{TN}}{\mathrm{TP}+\mathrm{TN}+\mathrm{FP}+\mathrm{FN}}$$

- Class Accuracy

Class accuracy evaluates the proportion of correctly predicted pixels for each class individually. This metric helps identify model performance across imbalanced or minority classes and was determined as follows:

(6) $${\mathrm{Class}\;\mathrm{Accuracy}}_{i}=\frac{{\mathrm{TP}}_{i}}{{\mathrm{TP}}_{i}+{\mathrm{FN}}_{i}}$$

where *i* denotes the class index.

- Intersection over Union (IoU)

IoU evaluates the spatial overlap between the predicted segmentation and ground truth by computing the ratio of their intersection to their union. IoU is particularly effective in assessing how well the model captures the extent of target regions and was determined as

(7) $$\mathrm{IoU}=\frac{\mathrm{TP}}{\mathrm{FP}+\mathrm{FN}+\mathrm{FN}}$$

- Weighted IoU

This metric is an extension of standard IoU that applies class-specific weights to address class imbalance. In vessel segmentation, where background regions vastly outnumber vessel pixels, this metric ensures that minority class performance is properly reflected in the evaluation. Weighted IoU was determined as follows:

(8) $$\mathrm{Weighted}\;\mathrm{IoU}=\sum_{c=1}^{C}w_{c}\cdot {\mathrm{IoU}}_{c}\mathrm{where}\;w_{c}=\frac{N_{c}}{\sum_{k=1}^{C}N_{k}}$$

where *C* is the number of classes, ${\mathrm{IoU}}_{c}$ is the IoU for class *c*, and $N_{c}$ is the number of pixels in class *c* in the ground truth.

- Mean Boundary F1 Score (MeanBFScore)

A boundary-based metric, MeanBFScore compares the predicted and ground truth object edges using the F1 score. This metric is especially relevant for medical segmentation tasks, such as retinal imaging, where accurate delineation of fine anatomic structures is critical. It was determined as

(9) $$\mathrm{BFScore}=\frac{2\;\cdot \;{\mathrm{Precision}}_{b}\;\cdot \;{\mathrm{Recall}}_{b}}{{\mathrm{Precision}}_{b}\;+{\mathrm{Recall}}_{b}}$$

where ${\mathrm{Precision}}_{b}$ and ${\mathrm{Recall}}_{b}$ are computed based on the distances between predicted and ground-truth boundaries within a tolerance (typically a few pixels). The final metric is the average BFScore over all images:

(10) $$\mathrm{MeanBFScore}=\frac{1}{N}\sum_{i=1}^{N}{\mathrm{BFScore}}_{i}$$

Together, these metrics provided a robust evaluation framework that considered pixel-level accuracy, object-level overlap, class imbalance, and boundary precision—key factors in determining segmentation performance in clinical applications.

## 4. Experiments

The performances of the CJA and PBNR models were assessed on the test datasets shown in Figure 2 and Figure 3. Inter-model evaluations were conducted, where the ground truth adjusted by each model underwent cross-testing. The findings derived from this analysis will yield insights into the efficacy of each correction methodology and their influence on segmentation performance across the datasets.

### 4.1. Experimental Settings

All experimental procedures were conducted utilizing MATLAB^®^ R2023a. The training and assessment methodologies were performed on a workstation furnished with an Intel^®^ Xeon^®^ E5-2689 0 @ 2.60 GHz central processing unit (Intel Corporation, Santa Clara, CA, USA), 32 GB of RAM, and an NVIDIA^®^ Tesla K40c graphics processing unit (NVIDIA Corporation, Santa Clara, CA, USA), which facilitated hardware acceleration for deep learning calculations.

### 4.2. Training Parameters

The neural network was developed utilizing retinal fundus images alongside their associated labeled segmentations. A stochastic gradient descent optimization algorithm was implemented with the following hyperparameters: 1000 training epochs, a batch size of two, a momentum coefficient of 0.9, a learning rate of 0.001, and a weight decay of 1 × 10^−4^. These parameters were meticulously chosen to facilitate stable convergence throughout the training process.

### 4.3. CJA Model Evaluation

To assess the impact of label refinement on segmentation performance, we conducted a retraining and evaluation experiment as the final stage of the CJA-based label correction pipeline. This step validated whether the improved labeling of evaluation data leads to better model performance.

**Figure 8:**
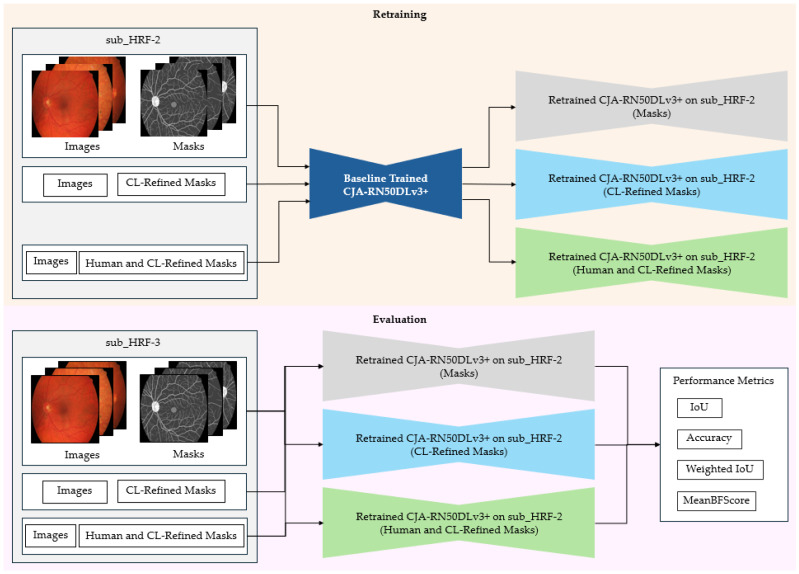
Retraining and evaluation with refined labels. This stage completes the CJA-based label refinement process by retraining the model using the corrected labels and evaluating its performance on clean test data. The goal is to assess the effectiveness of label correction in improving segmentation accuracy.

Figure 8 presents the retraining and evaluation process, where the baseline trained CJA-RN50DLv3+ model was trained on sub_HRF-2 under three distinct scenarios:

1. Original labels—the model was trained using the initial original labels without any correction, representing the baseline scenario.
2. CL-refined labels (Scenario 1)—the model was trained using labels refined solely through CL, based on the confident joint matrix derived from the original masks.
3. Human and CL-refined labels (Scenario 2)—the model was trained using labels refined through a combination of human correction and CL, representing the most reliable version of the ground truth.

These three trained models were then evaluated on the sub_HRF-3 dataset using both the original and corrected label sets to ensure a fair performance comparison. This experiment specifically aimed to assess how varying levels of label refinement affected the generalization capability of the model.

This setup enabled a comparative evaluation of model performances under different label quality conditions, demonstrating the effectiveness of CL-based refinement in improving training data and enhancing semantic segmentation accuracy.

Table 8 presents the segmentation performance across all model training configurations, demonstrating consistent improvements as the quality of the test set labels increased. The best results were achieved when the models were evaluated on the Scenario 2-refined test set (human and CL-refined masks). The model trained on CL-refined masks yielded the highest weighted IoU (0.95709), whereas the model trained on the human and CL-refined masks achieved the highest global accuracy (0.97697), IoU (0.91557), and MeanBFScore (0.98452). This configuration also provided a competitive class accuracy (0.95056), indicating strong performance across both the majority and minority classes.

These findings demonstrate that training with refined labels—particularly those corrected through a combination of human inspection and CL—could significantly enhance segmentation quality and model generalization. While global accuracy, IoU, and weighted IoU showed substantial improvements after label refinement, the MeanBFScore remained relatively stable, suggesting that the boundary-level precision improved only moderately. Since the highest metric values were achieved on the Scenario 2-refined sub_HRF-3 test set, the class-wise segmentation performances (accuracy, IoU, and MeanBFScore) of all three models were further evaluated exclusively on this refined version (Table 9).

As shown in Table 9, the background and retina classes returned consistently high scores across all models, indicating that they were relatively easier to segment. The fovea class benefited the most from label refinement, with accuracy improving from 0.8420 (original training) to 0.9423 and IoU from 0.8335 to 0.9272, highlighting the impact of label refinement on small or ambiguous structures. The vessel class also showed notable increases in both IoU and boundary precision, with the MeanBFScore exceeding 0.98 for all refined models, reflecting the improved localization of thin and complex structures. The optic disc class showed moderate but consistent gains in all metrics. These results confirmed that label refinement—particularly combining human and CL methods—could enhance not just overall performance but also class-specific segmentation quality, especially for challenging anatomic regions.

These refinements directly influenced both model training and evaluation. By reducing label noise and improving annotation quality, particularly in anatomically complex regions such as vessels and optic discs, the refined datasets enabled more precise learning of class boundaries. Models trained on these improved datasets exhibited stronger generalization and superior segmentation performance across all key metrics. This effect was especially evident under high-noise conditions, where poor label quality has a greater impact. The integration of CL with human review not only enhanced label reliability but also contributed to more stable benchmarking and fairer comparisons across different model configurations.

### 4.4. PBNR Model Evaluation

The images from the STARE dataset contained heterogeneous backgrounds. Setting the background intensity to zero in the preprocessing significantly improved the segmentation accuracy by mitigating background noise and enhancing vessel visibility. To assess the impact of label refinement using the PBNR method, the field of view provided a comparative retraining and evaluation experiment aimed at quantifying performance improvements when training with CL-refined labels versus original noisy annotations.

**Figure 9:**
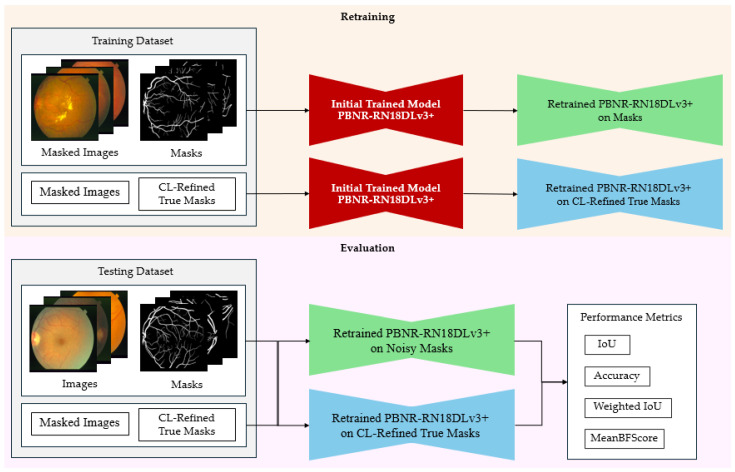
Retraining and evaluation with refined labels. This stage completes the PBNR-based label refinement process by retraining the model using pruned and corrected labels, and then evaluating its performance on a clean test dataset. The objective is to assess how effective the PBNR method is in improving segmentation accuracy by removing high-noise label instances.

Figure 9 shows the process of retraining and evaluating, where the PBNR-RN18DLv3+ model was retrained using the STARE training dataset under two conditions: (1) the original annotations, AH set, and (2) the refined AH labels created using the PBNR framework. Each model was evaluated on the same set of six AH test images using two conditions of test masks: (1) the original AH masks and (2) the CL-refined masks. This setup allowed us to isolate the effect of label quality on segmentation performance and analyze model generalization across varying annotation conditions.

Table 10 presents a comparative evaluation of the PBNR-RN18DLv3+ model trained on the original and CL-refined AH masks from the STARE dataset and tested on both original and CL-refined masks. When trained on the original AH labels, the model achieved a global accuracy of 0.96881 and an IoU of 0.79758 on the original test masks. Its performance improved when evaluated on CL-refined masks, reaching a 0.97021 global accuracy and 0.81508 IoU. Notably, class accuracy also increased from 0.85475 to 0.89097, and the weighted IoU improved from 0.94232 to 0.94598. The MeanBFScore showed a slight increase from 0.93542 to 0.93828, indicating stable boundary performance.

In contrast, the model trained on CL-refined AH masks consistently outperformed its counterpart. It achieved a 0.97092 global accuracy and 0.81811 IoU on the original test masks, and further improved to 0.97776 and 0.86297, respectively, on the CL-refined test masks. Class accuracy increased to 0.90188 and the weighted IoU to 0.95758, highlighting improved segmentation quality across both the vessel and background classes. The MeanBFScore also increased significantly, from 0.94609 to 0.96692, suggesting enhanced boundary precision under refined training and testing conditions.

Overall, these results confirmed that training with CL-refined labels could enhance model performances across all major metrics—particularly in IoU, class accuracy, and weighted IoU—while also contributing to improved boundary delineation. The findings underscored the effectiveness of CL-guided label correction in producing more accurate and robust semantic segmentation models for medical imaging.

### 4.5. Cross-Validation of Label Quality Between the CJA and PBNR Methods

Table 11 presents a performance comparison of the PBNR-RN18DLv3+ model trained on either the original or CL-refined AH masks from the STARE dataset, evaluated across three versions of the sub_HRF-3 test set: original masks, CL-refined masks, and human and CL-refined masks.

The model trained on the original masks achieved a global accuracy of 0.94871 and an IoU of 0.77975 when evaluated on the original test set. Its performance improved noticeably when evaluated on the CL-refined and human and CL-refined masks, with IoU increasing to 0.81452 and 0.81507, respectively. The MeanBFScore also increased to approximately 0.9546 in both cases, indicating improved boundary-level prediction. These results highlighted the significant impact of the test label quality on evaluation outcomes, even when the training data itself were not refined.

Conversely, the model trained on CL-refined masks consistently outperformed the original trained counterpart across all test sets. Even on the original test masks, it achieved better performance, returning an IoU of 0.79626, global accuracy of 0.94885, and a higher MeanBFScore of 0.95525. When evaluated on the CL-refined and human and CL-refined masks, its performances improved further, with the global accuracy exceeding 0.955, IoU around 0.812–0.813, and MeanBFScore peaking at approximately 0.965.

These findings indicate that refining training labels using CL could generate more accurate, generalizable, and robust segmentation models. Also, the improved performance across all test set variants reinforces the critical role of label quality not only in training but also in evaluation. The consistent gains observed with CL-refined masks confirm the value of CL in producing reliable annotations that advance the segmentation of medical images.

Table 12 compares the performance of the CJA-RN50DLv3+ model trained on three types of segmentation masks from the sub_HRF-2 dataset—original, CL-refined, and human and CL-refined—evaluated on both the original and CL-refined AH masks from the STARE dataset.

Overall, the model trained on CL-refined masks consistently delivered superior performances across nearly all metrics. When evaluated on CL-refined AH masks, it achieved the highest values for global accuracy (0.95595) and weighted IoU (0.91907), and, notably, the best MeanBFScore of 0.87515, indicating strong boundary delineation. Even when tested on original AH masks, the CL-trained model maintained robustness (MeanBFScore: 0.86262), suggesting effective generalization.

Interestingly, the model trained on the original masks also performed reasonably well, especially on CL-refined test masks (global accuracy, 0.95645; weighted IoU, 0.92089), implying that, despite label noise during training, the model retained some resilience and was still capable of producing competitive results under cleaner evaluation conditions.

In contrast, the model trained on human and CL-refined masks showed the weakest performances across both test sets. For example, it yielded a lower MeanBFScore (down to 0.81360 on original AH masks and 0.84084 on CL-refined masks), suggesting that the integration of manual corrections may have introduced inconsistencies or noise, especially when not guided by systematic approaches such as CL.

These findings highlight the critical role of systematic label refinement, particularly through CL, in improving segmentation performance. The combination of CL-refined labels in both training and evaluation settings produced the most reliable and accurate results, especially at the boundary level, underscoring the effectiveness of CL in enhancing model robustness and ensuring consistent benchmarking in medical image segmentation.

### 4.6. Computational Efficiency and Runtime Analysis

We evaluated the runtime performance of the proposed models using MATLAB^®^ R2023a on a workstation equipped with an Intel^®^ Xeon^®^ E5-2689 0 @ 2.60 GHz CPU, 32 GB of RAM, and an NVIDIA^®^ Tesla K40c GPU, which provided hardware acceleration for deep learning computations. All experiments were conducted using retinal images at a resolution of 1024 × 1024 pixels.

Table 13 presents the average inference times of the models, showing a time of 0.8355 s per image for the baseline DeeplabV3+ResNet-50 model, and a time of 0.8344 s per image for the DeeplabV3+ResNet-50 + CL (Scenario 1) variant. The DeeplabV3+ResNet-18 model recorded an average inference time of 0.4413 s per image, which the CL-integrated version (DeeplabV3+ResNet-18 + CL (Scenario 1)) reduced marginally to 0.4410 s.

The estimated computational cost, measured in Giga Floating Point Operations (GFLOPs), remained unchanged across each backbone: 546.27 GFLOPs for ResNet-50-based models and 329.14 GFLOPs for ResNet-18-based models. Throughput was calculated by dividing GFLOPs by the corresponding inference time, resulting in 654.69 GOPSs for DeeplabV3+ResNet-50 + CL (Scenario 1) and 746.32 GOPSs for DeeplabV3+ResNet-18 + CL (Scenario 1).

Model size also remained unaffected by CL integration, with 175.6 MB for the ResNet-50-based models and 82.4 MB for the ResNet-18-based models.

These results confirmed that incorporating CL introduced no additional computational overhead during inference. The high throughput and relatively compact model sizes demonstrated the practicality of these models for real-time deployment in clinical settings, particularly in embedded or resource-constrained environments. These findings align with recent research into efficient deep learning solutions for ocular disease diagnosis [38,39].

## 5. Discussion

The implementation of the CJA and PBNR frameworks highlighted the practical utility of CL techniques in identifying and correcting labeling errors in medical image segmentation. The CJA-RN50DLv3+ model was trained and fine-tuned using the corrected DRIVE and CHASE-DB1 datasets and evaluated on the HRF dataset. This evaluation enabled the construction of a confident joint matrix that revealed inconsistencies between observed and true labels. Images were then enhanced through CLAHE preprocessing to improve the clarity of anatomic details for more accurate human verification. The final model improved labeling precision by efficiently detecting false positives and false negatives, especially in the vessel class. The enhancement of segmentation performance was validated through probability-based thresholding.

The PBNR-RN18DLv3+ model presents a structured strategy for identifying class-dependent label noise in retinal vessel segmentation. A prioritization mechanism, based on normalized margin scores, ranks suspected mislabeled pixels by uncertainty and the model leverages predictions with associated confidence scores to isolate mislabeled pixels and estimate noise rates across different classes. When applied to the STARE dataset, this process revealed significant discrepancies between the observed and reference annotations that demonstrated the ability of CL to uncover latent labeling errors. While not all detected errors were confirmed, the substantial overlap with known annotation disagreements supported the reliability of the noise detection strategy. The refinement of STARE annotations based on these insights enabled the construction of a cleaner training subset. The improved label integrity of the subset enhanced segmentation quality, reinforcing the practical value of PBNR.

The CJA and PBNR model evaluations presented in Section 4.3 and Section 4.4, respectively, highlighted the critical role of label refinement strategies based on CL. Both semantic segmentation models showed improved accuracy and generalizability for retinal image analysis. Across all experiments, including the PBNR-based evaluation summarized in Table 10, models trained and evaluated using CL-refined segmentation masks consistently outperformed those trained on the original annotations. The PBNR-RN18DLv3+ model, especially when trained on CL-refined labels, showed clear improvements across the key evaluation metrics when tested on both original and corrected annotations. These improvements were particularly notable in the segmentation of retinal structures such as vessels and the fovea, which are highly sensitive to annotation noise. This study supports previous studies that demonstrated the negative impact of noisy or inaccurate labels on medical image segmentation tasks [4,5,9,11,18,19,20,21]. Overall, the results of the present study demonstrate that CL-guided label correction enables the development of more accurate, reliable, and robust segmentation models.

Our cross-dataset evaluations in Section 4.5 further substantiated the role of CL in guiding label refinement for medical image segmentation. As shown in Table 11, training on CL-refined labels yielded a better boundary quality, as indicated by the highest MeanBFScore. In contrast, models trained on original labels returned slightly better scores for global accuracy and IoU when evaluated on the same CL-refined test set. This result suggests that the choice of annotation involves a trade-off. It may be that CL enhances structural precision, while original labels better preserve pixel-level consistency. The impact of label refinement became more apparent when the model was trained on the original STARE-AH masks and tested on different versions of sub_HRF-3. Even without changing the training data, replacing noisy test masks with CL-refined masks significantly improved the MeanBFScore. Further gains occurred when both training and test masks were refined. These results confirmed that, even without architectural changes, CL improved model generalization and segmentation reliability, reinforcing its value in clinical settings [1,18,19,20,21]. The effect of integrating human corrections was revealed in the MeanBFScore values (Table 12). While combining human and CL-based refinements may seem beneficial, the approach did not consistently produce better outcomes than purely CL-guided corrections. Specifically, the CJA-RN50DLv3+ model trained only on CL-refined labels from sub_HRF-2 achieved the highest MeanBFScore of 0.87515 when evaluated on CL-refined AH masks. In contrast, the same model trained on human and CL-refined labels returned a MeanBFScore of 0.84084. This result suggests that unstructured or inconsistent human corrections may reduce segmentation precision by introducing label variability at object boundaries. These results align with the conclusions of Zhang et al. [32], who highlighted the importance of the structured characterization of labeling errors to improve annotation quality and model reliability.

In the present investigation, the comparative analysis of the proposed methodology was primarily conducted against a baseline DeepLabV3+ model trained on the original noisy dataset, thereby establishing a foundational performance benchmark. While this benchmark could serve as a critical reference point, it is recognized that contemporary state-of-the-art methodologies for addressing label noise, including Co-teaching [40], MentorNet [41] and noise-robust loss functions such as generalized cross-entropy loss [42], represent alternative strategies that warrant a thorough examination. These methodologies have shown their efficacy in alleviating the detrimental effects of noisy labels across a diverse array of domains. Nonetheless, owing to limitations in computational resources and the scope of the current investigation, direct comparisons with these techniques could not be drawn from the present experiments. This is regarded as a limitation, and more current comparative analyses will be pursued in future research endeavors to provide a more exhaustive and rigorous assessment of the proposed framework.

Taken together, these findings underscore the critical importance of addressing label noise in medical image segmentation. Our cross-dataset evaluations demonstrated that models trained with CL-refined labels not only generalize more effectively to unseen data but also benefit from more reliable performance assessments when evaluated against corrected ground truth masks. This finding reinforces the crucial role of high-quality test sets in ensuring trustworthy and stable benchmarking outcomes. The integration of CL, especially when complemented with human verification, provides a scalable and robust framework for improving annotation integrity. This dual approach strengthens both the training and evaluation phases, directly responding to concerns raised in recent studies regarding the destabilizing impact of label errors in test sets [20]. Future work should explore combining CL with other label quality estimation techniques, extending the application of CL to multi-class and multi-modal medical imaging tasks—such as OCT and MRI—and evaluating its influence on clinical diagnostic accuracy and decision-making.

The precise delineation of retinal blood vessels holds significant clinical implications within the field of ophthalmology, particularly concerning conditions such as DR and glaucoma. In relation to DR, accurate vascular mapping is crucial for the identification of microaneurysms, hemorrhages, and neovascularization, pathological indicators that often lie adjacent to or disrupt vascular architectures. Improved segmentation facilitates more dependable lesion localization and bolsters automated grading systems for DR. In the case of glaucoma, the vascular morphology surrounding the optic disc plays a vital role in the estimation of the cup-to-disc ratio and the recognition of aberrant vascular patterns, which are essential for a prompt diagnosis. Furthermore, clinical evaluations take into account quantitative biomarkers obtained from vessel segmentation, including tortuosity, caliber, and branching angles. Consequently, the enhancement of vessel segmentation through label correction techniques, such as CL, not only augments model efficacy but also fortifies the reliability, interpretability, and clinical relevance of automated screening systems.

In summary, this study validates the incorporation of CL-based label refinement as a foundational element in reliable semantic segmentation pipelines. When paired with selective human oversight, CL-guided corrections significantly enhance the consistency and accuracy of both training and testing datasets, effectively mitigating the widespread issue of noisy ground truth annotations in medical image analysis.

## 6. Limitations

Despite the overall effectiveness of CL in improving label quality and enhancing segmentation performance, this study identifies several limitations that warrant further investigation. A primary concern arises from the thresholding mechanism employed during the label correction process, which can lead to the misclassification of pixels, even when the model exhibits high confidence in its predictions.

**Figure 10:**
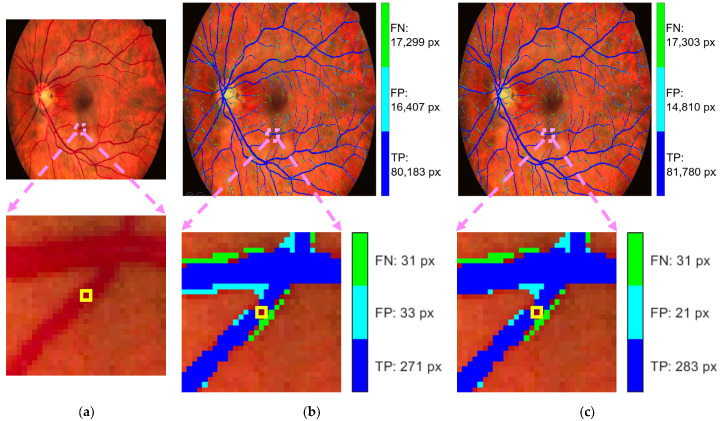
Example from Image1024-02_g showing a misclassified pixel, highlighted in the yellow box across all subfigures, caused by the thresholding mechanism in the CL-based label refinement process for the vessel class: (**a**) enhanced retinal image; (**b**) Scenario 1—overlay of true positives, false positives, and false negatives; and (**c**) Scenario 2—overlay of true positives, false positives, and false negatives.

Figure 10 presents a visual example from Image1024-02_g, in which a vessel pixel—highlighted in a yellow box across all subfigures—is misclassified by the CJA model due to the fixed thresholding mechanism used in the CL-based label refinement process. Table 14 provides the predicted probability scores for this specific pixel across all classes, along with the corresponding class-specific threshold values that were used to determine whether the prediction should be retained or rejected as out of the distribution.

The pixel belonged to a vessel but had a predicted probability of 0.9306, which fell short of the predefined vessel-specific threshold of 0.9744. The pixel was incorrectly flagged as out of the distribution. This case exemplifies how fixed thresholds may fail to capture the true prediction confidence, particularly in regions near class decision boundaries.

**Figure 11:**
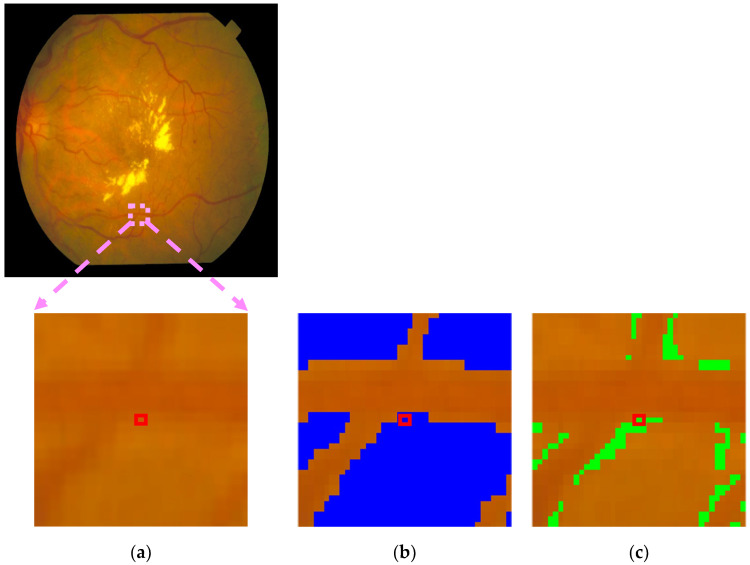
Example from im0001 showing a misclassified pixel, highlighted in the red box across all subfigures: (**a**) original retinal image; (**b**) background ground truth from AH (highlighted in blue); and (**c**) false negatives (highlighted in green).

Figure 11 presents a visual example from image im0001, in which a vessel pixel is highlighted in a red box across all subfigures. Figure 11b shows that the pixel was originally labeled as background by the PBNR model. The PBNR-RN18DLv3+ model predicted this pixel as an undefined or out-of-distribution class because its predicted probability scores as vessel (0.8511) or background (0.1489) (Table 15) did not exceed the respective threshold values (0.9755 for vessel and 0.9982 for background). Figure 11c (see also Figure 7c and Table 6) indicates that this pixel classification is a false negative. This result means that CL failed to identify this mislabeled pixel, indicating a limitation in its detection capability.

These issues stem from (1) the ambiguity in predicted probabilities near boundaries between classes and (2) the inflexibility of fixed thresholds that do not adapt to local context or uncertainty. Such challenges are critical in medical image segmentation tasks involving fine, complex anatomical structures like retinal vessels.

To address these limitations within this study, we incorporated adaptive thresholding strategies that dynamically adjust threshold values based on local prediction distributions, which helped reduce false rejections. In addition, we applied spatial continuity constraints informed by vessel connectivity heuristics to improve correction robustness in boundary-sensitive regions. These refinements proved effective in mitigating threshold-related misclassifications and contributed to the overall improvement of the annotation quality. The integration of these techniques within our CL-based framework demonstrates its scalability and robustness in clinical retinal image analysis, particularly under conditions of label uncertainty and structural complexity.

Lastly, another prominent limitation of this study involved the domain shift and image quality differences between the training (HRF) and testing (STARE) datasets. Although all images were resized to 1024 × 1024 pixels, the original STARE images possessed a significantly lower visual quality, particularly in sharpness, contrast, and anatomical details. These deficiencies most likely impaired the segmentation performance, especially at the boundaries of small or ambiguous structures such as vessels and the optic disc. As shown in Table 12, all models, including the best-performing model trained on CL-refined labels, experienced a decrease in the MeanBFScore under this cross-dataset evaluation. The decline in performance highlighted the importance of domain consistency as well as image fidelity when applying label correction strategies across heterogeneous datasets.

## 7. Conclusions

The rectification of label noise via confident learning and subsequent human refinement markedly enhances the efficacy of semantic segmentation in retinal fundus images. Across different datasets and evaluative metrics, models that were trained on refined labels consistently exhibited superior performances compared with those trained on noisy labels. Significantly, CL-refined models demonstrated enhanced generalization capabilities to previously unseen datasets and yielded more reliable evaluations when assessed against corrected ground truths, thereby underscoring the pivotal importance of the test set quality in facilitating equitable and dependable benchmarking.

The innovative approach presented integrates algorithmic label refinement with rigorous human verification, resulting in highly accurate segmentation not only of prominent anatomic structures but also of subtle pathological regions that may otherwise be overlooked. This dual methodology highlights the critical importance of scalable label correction frameworks in the realm of medical image analysis, demonstrating their capacity to enhance the precision and reliability of diagnostic imaging. Furthermore, the findings emphasize the necessity of prioritizing annotation quality within both training and evaluation pipelines, as high-quality annotations are essential for developing robust machine learning models that can effectively interpret complex medical data. By ensuring meticulous attention to detail in the labeling process, significant improvements can be made to the overall accuracy of automated systems deployed to analyze and identify medical conditions, ultimately leading to better patient outcomes.

## Figures and Tables

**Table 1:** Summary of the retinal fundus image datasets used in this study.

Dataset	Description	Image Count	Resolution (Pixels)	Camera Used	Unique Features
CHASE_DB1	Child Health and Heart Studies in England (CHASE) provided with vessel ground truth in the first database (CHASE_DB1).	28	1280 × 960	Nidek NM-200D, 35 °FOV	Ground truths were annotated by two experts, focusing on a pediatric population.
DRIVE	Digital Retinal Images for Vessel Extraction (DRIVE) acquired for DR screening.	40	768 × 584	Canon CR5 non-mydriatic 3CCD, 45 °FOV	Ground truths were annotated by two experts for trained images and three experts for test images.
HRF	High-Resolution Fundus (HRF) designed 15 images for each group of healthy, DR, and glaucoma patients.	45	3504 × 2336	Canon CR-1 fundus camera, 45 °FOV	Ground truths were annotated by experts in the retinal image analysis field.
STARE	Structured Analysis of the Retina (STARE), 11 images showed pathologies.	20	605 × 700	TRV-50 Topcon, 35 °FOV	Ground truths with different levels of small vessel segmentations were annotated by two experts.

**Table 2:** Confidence thresholds employed for the identification of labeling inaccuracies within five anatomic structures of the retina.

Class	Threshold Value
Background	0.9990
Retina	0.9969
Fovea	0.9982
Vessel	0.9744
Optic Disc	0.9877

**Table 3:** Confident joint matrix pertaining to Image1024-02_g for the purpose of detecting labeling inaccuracies via off-diagonal elements.

True Label (y*)	Observed Label (*ỹ*)				
	Background	Retina	Fovea	Vessel	Optic Disc
Background	96,829	9	0	1	0
Retina	10	775,257	848	16,686	883
Fovea	0	205	4699	1	0
Vessel	4	14,137	0	81,780	669
Optic Disc	0	65	0	615	7426

**Table 4:** Confidence thresholds employed for the designation of error detection within the background and vessel class.

Class	Threshold Value
Background (1)	0.9982
Vessel (2)	0.9755

**Table 5:** Confident joint matrix for im0001: an estimated correlation between the observed labels and the true labels.

True Label (y*)	Observed Label (*ỹ*)	
	Background (1)	Vessel (2)
**Background (1)**	904,136	5981
**Vessel (2)**	23,960	68,862

**Table 6:** Confusion matrix comparing the observed issue mask (from CL) and the true issue mask (from AH–VK disagreement).

True Issue	Observed Issue	
	True	False
True	12,354 (TP)	25,731 (FN)
False	17,578 (FP)	992,904 (TN)

**Table 7:** Top 10 ranked label errors detected by CL in im0001 using normalized margin scores.

Observation Index	Score	Observed Label	True Label *	True Issue *
41,485	0	2	1	1
41,486	0	2	1	1
47,862	0	2	2	0
47,864	0	2	1	1
47,865	0	2	1	1
47,866	0	2	1	1
47,867	0	2	1	1
47,868	0	2	1	1
47,869	0	2	1	1
47,870	0	2	1	1

* “True Label” refers to the actual label based on the ground truth provided by the VK annotations. “True Issue” indicates whether the pixel is a true labeling error, determined by discrepancies between the annotations from AH and VK shown in Figure 7b.

**Table 8:** Performance comparison of CJA-RN50DLv3+ trained with different types of segmentation masks and evaluated across various test set versions.

Training Dataset	Testing Dataset	Global Accuracy	Class Accuracy	IoU	Weighted IoU	MeanBFScore
Original Masks	Original Masks	0.95090	0.87292	0.81478	0.91098	0.95108
	CL-Refined Masks	0.96675	0.91852	0.87223	0.93942	0.97517
	Human and CL-Refined Masks	0.96682	0.91855	0.87243	0.93953	0.97516
CL-Refined Masks	Original Masks	0.95447	0.88526	0.82197	0.9166	0.95367
	CL-Refined Masks	0.97699	0.95149	0.91398	0.95694	0.98064
	Human and CL-Refined Masks	0.97708	0.95152	0.91421	0.95709	0.98064
Human and CL- Refined Masks	Original Masks	0.95389	0.88248	0.81956	0.91532	0.95254
	CL-Refined Masks	0.97687	0.95051	0.91532	0.95655	0.98454
	Human and CL-Refined Masks	0.97697	0.95056	0.91557	0.95672	0.98452

**Table 9:** Class-wise segmentation performance of CJA-RN50DLv3+ on the Scenario 2-refined sub_HRF-3 test set.

Metric	Training Dataset	Background	Retina	Fovea	Vessel	Optic Disc
Accuracy	Original Masks	0.9988	0.9752	0.8420	0.8800	0.8967
	CL-Refined Masks	0.9989	0.9829	0.9339	0.9156	0.9263
	Human and CL-Refined Masks	0.9984	0.9844	0.9423	0.9023	0.9255
IoU	Original Masks	0.9953	0.9601	0.8335	0.7310	0.8423
	CL-Refined Masks	0.9959	0.9723	0.9142	0.8037	0.8850
	Human and CL-Refined Masks	0.9958	0.9721	0.9272	0.8009	0.8819
MeanBFScore	Original Masks	0.9995	0.9682	0.9487	0.9731	0.9863
	CL-Refined Masks	0.9997	0.9820	0.9480	0.9856	0.9880
	Human and CL-Refined Masks	0.9995	0.9808	0.9727	0.9849	0.9848

**Table 10:** Performance comparison of PBNR-RN18DLv3+ trained with original vs. CL-refined AH masks from STARE and evaluated on original and CL-refined test masks.

Training Dataset	Testing Dataset	Global Accuracy	Class Accuracy	IoU	Weighted IoU	MeanBFScore
Original AH Masks	Original Masks	0.96881	0.85475	0.79758	0.94232	0.93542
	CL-Refined Masks	0.97021	0.89097	0.81508	0.94598	0.93828
CL-Refined AH Masks	Original Masks	0.97092	0.85189	0.81811	0.94431	0.94609
	CL-Refined Masks	0.97776	0.90188	0.86297	0.95758	0.96692

**Table 11:** Performance comparison of PBNR-RN18DLv3+ trained with original vs. CL-refined AH masks from STARE and evaluated across various test set versions of sub_HRF-3.

Training Dataset (AH)	Testing Dataset (sub-HRF-3)	Global Accuracy	Class Accuracy	IoU	Weighted IoU	MeanBFScore
Original Masks	Original Masks	0.94871	0.86066	0.77975	0.90805	0.93072
	CL-Refined Masks	0.96043	0.90840	0.81452	0.92956	0.95465
	Human and CL-Refined Masks	0.96055	0.90857	0.81507	0.92974	0.95461
CL-Refined Masks	Original Masks	0.94885	0.91071	0.79626	0.91119	0.95525
	CL-Refined Masks	0.95575	0.95275	0.81231	0.92459	0.96537
	Human and CL-Refined Masks	0.95589	0.95295	0.81291	0.92480	0.96534

**Table 12:** Performance comparison of CJA-RN50DLv3+ trained with different types of segmentation sub_HRF-2 masks and evaluated on original and CL-refined AH masks from STARE.

Training Dataset (sub_HRF-2)	Testing Dataset (AH)	Global Accuracy	Class Accuracy	IoU	Weighted IoU	MeanBFScore
Original Masks	Original Masks	0.95496	0.81879	0.73470	0.92084	0.84532
	CL-Refined Masks	0.95645	0.81726	0.75252	0.92089	0.86274
CL-Refined Masks	Original Masks	0.95590	0.80403	0.73144	0.92131	0.86262
	CL-Refined Masks	0.95595	0.79957	0.74339	0.91907	0.87515
Human and CL- Refined Masks	Original Masks	0.94970	0.79046	0.70731	0.91231	0.81360
	CL-Refined Masks	0.94926	0.78570	0.71769	0.90908	0.84084

**Table 13:** Computational performances of baseline and CL-integrated models using DeeplabV3+ with ResNet-50 and ResNet-18 backbones. Throughput is measured in GOPSs (Giga Operations Per Second). GFLOPs = Giga Floating Point Operations; MB = megabytes.

Model	GFLOPs	Inference Time (s/image)	Throughput (GOPS)	Model Size (MB)
DeeplabV3+ResNet-50	546.27	0.8355	653.83	175.6
DeeplabV3+ResNet-50 + CL (Scenario 1)	546.27	0.8344	654.69	175.6
DeeplabV3+ResNet-18	329.14	0.4413	745.80	82.4
DeeplabV3+ResNet-18 + CL (Scenario 1)	329.14	0.4410	746.32	82.4

**Table 14:** Predicted probability scores and corresponding class-specific thresholds for the misclassified vessel pixel shown in Figure 10.

Class	Predicted Probability Score	Threshold Score
Background	0.000004	0.9990
Retina	0.0694	0.9969
Fovea	0.000003	0.9982
Vessel	0.9306	0.9744
Optic Disc	0.000002	0.9877

**Table 15:** Predicted probability scores and corresponding class-specific thresholds for the misclassified vessel pixel shown in Figure 11.

Class	Predicted Probability Score	Threshold Score
Background	0.1489	0.9982
Vessel	0.8511	0.9755

## Data Availability

The original contributions presented in the study are included in the article; further inquiries can be directed to the corresponding author.

## Abbreviations

The following abbreviations are used in this manuscript:

DR	Diabetic Retinopathy
CL	Confident Learning
CJA	Confident Joint Analysis
PBNR	Prune by Noise Rate
Acc	Accuracy
IoU	Intersection over Union
MeanBFScore	Mean Boundary F1 Score
FOV	Field of View
TP	True Positive
FP	False Positive
FN	False Negative
TN	True Negative
px	Pixel

## References

[1] Ikram A., Imran A., Li J., Alzubaidi A., Fahim S., Yasin A., Fathi H. (2024). A systematic review on fundus image-based diabetic retinopathy detection and grading: Current status and future directions. IEEE Access.

[2] Del Pinto R., Mulè G., Vadalà M., Carollo C., Cottone S., Agabiti Rosei C., De Ciuceis C., Rizzoni D., Ferri C., Muiesan M.L. (2022). Arterial hypertension and the hidden disease of the eye: Diagnostic tools and therapeutic strategies. Nutrients.

[3] Camara J., Neto A., Pires I.M., Villasana M.V., Zdravevski E., Cunha A. (2022). A comprehensive review of methods and equipment for aiding automatic glaucoma tracking. Diagnostics.

[4] Patton N., Aslam T.M., MacGillivray T., Deary I.J., Dhillon B., Eikelboom R.H., Yogesan K., Constable I.J. (2006). Retinal image analysis: Concepts, applications and potential. Prog. Retin. Eye Res..

[5] Abràmoff M.D., Garvin M.K., Sonka M. (2010). Retinal imaging and image analysis. IEEE Rev. Biomed. Eng..

[6] Long J., Shelhamer E., Darrell T. (2014). Fully convolutional networks for semantic segmentation. arXiv.

[7] Li Z., Jia M., Yang X., Xu M. (2021). Blood vessel segmentation of retinal image based on dense-U-Net network. Micromachines.

[8] Chen L., Zhu Y., Papandreou G., Schroff F., Adam H. (2018). Encoder-Decoder with Atrous Separable Convolution for Semantic Image Segmentation. Proceedings of the European Conference on Computer Vision (ECCV).

[9] Staal J., Abràmoff M.D., Niemeijer M., Viergever M.A., Van Ginneken B. (2004). Ridge-Based Vessel Segmentation in Color Images of the Retina. IEEE Trans. Med. Imaging.

[10] Hoover A., Kouznetsova V., Goldbaum M. (2000). Locating Blood Vessels in Retinal Images by Piecewise Threshold Probing of a Matched Filter Response. IEEE Trans. Med. Imaging.

[11] Fraz M.M., Remagnino P., Hoppe A., Uyyanonvara B., Rudnicka A.R., Owen C.G., Barman S.A. (2012). An ensemble classification-based approach applied to retinal blood vessel segmentation. IEEE Trans. Biomed. Eng..

[12] Budai A., Bock R., Maier A., Hornegger J., Michelson G. (2013). Robust Vessel Segmentation in Fundus Images. Int. J. Biomed. Imaging.

[13] Jin K., Huang X., Zhou J., Li Y., Yan Y., Sun Y., Zhang Q., Wang Y., Ye J. (2022). FIVES: A Fundus Image Dataset for Artificial Intelligence Based Vessel Segmentation. Sci. Data.

[14] Lyu X., Cheng L., Zhang S. (2022). The RETA Benchmark for Retinal Vascular Tree Analysis. Sci. Data.

[15] Lepetit-Aimon G., Playout C., Boucher M.C., Duval R., Brent M.H., Cheriet F. (2024). MAPLES-DR: MESSIDOR Anatomical and Pathological Labels for Explainable Screening of Diabetic Retinopathy. Sci. Data.

[16] Guo E., Wang Z., Zhao Z., Zhou L. (2025). Imbalanced Medical Image Segmentation with Pixel-dependent Noisy Labels. arXiv.

[17] Pranto T.H., Noman A.A., Noor A., Deepty U.H., Rahman R.M. (2022). Effect of Label Noise on Multi-Class Semantic Segmentation: A Case Study on Bangladesh Marine Region. Appl. Artif. Intell..

[18] Xu Z., Lu D., Luo J., Wang Y., Yan J., Ma K., Zheng Y., Tong R.Y. (2022). Anti-Interference from Noisy Labels: Mean-Teacher-Assisted Confident Learning for Medical Image Segmentation. IEEE Trans. Med. Imaging.

[19] Tajbakhsh N., Jeyaseelan L., Li Q., Chiang J.N., Wu Z., Ding X. (2020). Embracing Imperfect Datasets: A Review of Deep Learning Solutions for Medical Image Segmentation. Med. Image Anal..

[20] Northcutt C.G., Athalye A., Mueller J. (2021). Pervasive Label Errors in Test Sets Destabilize Machine Learning Benchmarks. arXiv.

[21] Karimi D., Dou H., Warfield S.K., Gholipour A. (2020). Deep Learning with Noisy Labels: Exploring Techniques and Remedies in Medical Image Analysis. Med. Image Anal..

[22] Wang Y., Luo L., Wu M., Wang Q., Chen H. (2025). Learning robust medical image segmentation from multi-source annotations. Med. Image Anal..

[23] Angluin D., Laird P. (1988). Learning from Noisy Examples. Mach. Learn..

[24] Northcutt C.G., Jiang L., Chuang I.L. (2021). Confident Learning: Estimating Uncertainty in Dataset Labels. J. Artif. Intell. Res..

[25] Northcutt C.G. (2021). Confident Learning for Machines and Humans. Doctoral Dissertation.

[26] Hao P., Shi K., Tian S., Wu F. (2023). Uncertainty-aware iterative learning for noisy-labeled medical image segmentation. IET Image Process..

[27] Tan J.H., Acharya U.R., Bhandary S.V., Chua K.C., Sivaprasad S. (2017). Segmentation of Optic Disc, Fovea and Retinal Vasculature Using a Single Convolutional Neural Network. J. Comput. Sci..

[28] Alsayat A., Elmezain M., Alanazi S., Alruily M., Mostafa A.M., Said W. (2023). Multi-layer Preprocessing and U-Net with Residual Attention Block for Retinal Blood Vessel Segmentation. Diagnostics.

[29] Liu R., Pu W., Nan H., Zou Y. (2023). Retina image segmentation using the three-path Unet model. Sci. Rep..

[30] Lad V., Mueller J. (2023). Estimating Label Quality and Errors in Semantic Segmentation Data via Any Model. arXiv.

[31] Zhang L., Tanno R., Xu M.C., Jin C., Jacob J., Ciccarelli O., Barkhof F., Alexander D.C. (2021). Disentangling Human Error from Ground Truth in Segmentation of Medical Images. Advances in Neural Information Processing Systems, Proceedings of the NeurIPS 2020, Vancouver, BC, Canada, 6–12 December 2020.

[32] Zhang M., Gao J., Lyu Z., Zhao W., Wang Q., Ding W., Wang S., Li Z., Cui S., Martel A.L. (2020). Characterizing Label Errors: Confident Learning for Noisy-Labeled Image Segmentation. Medical Image Computing and Computer-Assisted Intervention—MICCAI 2020, Proceedings of the 23rd International Conference, Lima, Peru, 4–8 October 2020.

[33] Shi J., Wu J. (2021). Distilling Effective Supervision for Robust Medical Image Segmentation with Noisy Labels. Medical Image Computing and Computer-Assisted Intervention—MICCAI 2021, Part I.

[34] Weng T., Shen Y., Jin K., Wang Y., Cheng Z., Li Y., Zhang G., Wang S. (2024). Enhancing point annotations with superpixel and confident learning guided for improving semi-supervised OCT fluid segmentation. Biomed. Signal Process. Control.

[35] Targ S., Almeida D., Lyman K. (2016). Resnet in Resnet: Generalizing Residual Architectures. arXiv.

[36] Reza A.M. (2004). Realization of the Contrast Limited Adaptive Histogram Equalization (CLAHE) for Real-Time Image Enhancement. J. VLSI Signal Process. Syst. Signal Image Video Technol..

[37] Image Labeler Documentation. https://www.mathworks.com/help/vision/ref/.

[38] Al Jbaar M.A., Dawwd S.A. (2023). DCNN-based embedded models for parallel diagnosis of ocular diseases. East.-Eur. J. Enterp. Technol..

[39] Al Jbaar M.A., Dawwd S.A. (2023). SIMD implementation of deep CNNs for myopia detection on a single-board computer system. East.–Eur. J. Enterp. Technol..

[40] Han B., Yao Q., Yu X., Niu G., Xu M., Hu W., Tsang I.W., Sugiyama M. (2018). Co-teaching: Robust training of deep neural networks with extremely noisy labels. Adv. Neural Inf. Process. Syst..

[41] Jiang L., Zhou Z., Leung T., Li L.J., Li F.-F. MentorNet: Learning Data-Driven Curriculum for Very Deep Neural Networks on Corrupted Labels. Proceedings of the 35th International Conference on Machine Learning (ICML).

[42] Zhang Z., Sabuncu M.R. (2018). Generalized cross entropy loss for training deep neural networks with noisy labels. Adv. Neural Inf. Process. Syst..

